# Natural Selection Promotes Antigenic Evolvability

**DOI:** 10.1371/journal.ppat.1003766

**Published:** 2013-11-14

**Authors:** Christopher J. Graves, Vera I. D. Ros, Brian Stevenson, Paul D. Sniegowski, Dustin Brisson

**Affiliations:** 1 University of Pennsylvania, Philadelphia, Pennsylvania, United States of America; 2 University of Kentucky, Lexington, Kentucky, United States of America; Oxford University, United Kingdom

## Abstract

The hypothesis that evolvability - the capacity to evolve by natural selection - is itself the object of natural selection is highly intriguing but remains controversial due in large part to a paucity of direct experimental evidence. The antigenic variation mechanisms of microbial pathogens provide an experimentally tractable system to test whether natural selection has favored mechanisms that increase evolvability. Many antigenic variation systems consist of paralogous unexpressed ‘cassettes’ that recombine into an expression site to rapidly alter the expressed protein. Importantly, the magnitude of antigenic change is a function of the genetic diversity among the unexpressed cassettes. Thus, evidence that selection favors among-cassette diversity is direct evidence that natural selection promotes antigenic evolvability. We used the Lyme disease bacterium, *Borrelia burgdorferi*, as a model to test the prediction that natural selection favors amino acid diversity among unexpressed *vls* cassettes and thereby promotes evolvability in a primary surface antigen, VlsE. The hypothesis that diversity among *vls* cassettes is favored by natural selection was supported in each *B. burgdorferi* strain analyzed using both classical (dN/dS ratios) and Bayesian population genetic analyses of genetic sequence data. This hypothesis was also supported by the conservation of highly mutable tandem-repeat structures across *B. burgdorferi* strains despite a near complete absence of sequence conservation. Diversification among *vls* cassettes due to natural selection and mutable repeat structures promotes long-term antigenic evolvability of VlsE. These findings provide a direct demonstration that molecular mechanisms that enhance evolvability of surface antigens are an evolutionary adaptation. The molecular evolutionary processes identified here can serve as a model for the evolution of antigenic evolvability in many pathogens which utilize similar strategies to establish chronic infections.

## Author Summary

The hypothesis that natural selection shapes the ability of a population to evolve is highly controversial due primarily to a paucity of empirical evidence. However, the ability to constantly and rapidly evolve may be selectively adaptive in pathogens due to the constantly changing environment created by the vertebrate immune response. The Lyme disease bacterium, *Borrelia burgdorferi*, evades immune detection through a system consisting of numerous unexpressed ‘cassette’ sequences that alter the expressed protein via recombination. Importantly, the potential for evolutionary change in the expressed protein, and thus its ability to repeatedly escape a directed immune response, is correlated with the diversity among the cassettes. We analyzed genetic data from diverse *B. burgdorferi* strains to test the hypothesis that natural selection favors diversity among the unexpressed cassettes in order to promote evolvability. The data provide significant evidence of natural selection acting to increase among-cassette diversity, but only in regions that are targeted by immune antibodies when expressed. Further, these antigenically-important regions contain mutation-prone DNA sequence structures that are conserved despite high levels of sequence divergence among strains. The empirical evidence of selection favoring mutation-prone sequences and favoring among-cassette diversity supports the hypothesis that natural selection can shape the evolvability of populations.

## Introduction

The ability of a biological trait to evolve by natural selection, or evolvability, varies substantially among species, among populations within species, and even among traits within populations. The hypothesis that differences in evolvability result from past natural selection acting on the ability to evolve, however, remains highly controversial for two primary reasons [1], [2], [3]. First, evolvability is a population-level phenotype and thus must be favored by the relatively weak forces generated by natural selection at the population level [4]. Second, selection on evolvability suggests the unlikely scenario that natural selection has the evolutionary foresight to adapt a population to future environmental contingencies [1]. These issues complicate the interpretation of studies which suggest that differences in evolvability arise as a result of natural selection [5], [6], [7], [8], [9].

The major objections to the evolvability-as-adaptation hypothesis lose force when applied to many microbial pathogens as a result of two biological features of these organisms. First, microbial pathogen cells within a host are often nearly clonal such that the fitness interests of individuals and groups are closely aligned [10], [11]. The exceedingly high degree of genetic relatedness among individuals in a host has resulted in compelling evidence of selection on population-level traits that are vital to the life-histories of microbial pathogens [10], [12], [13], [14]. Second, the history of consistent environmental uncertainty caused by the dynamic immune response is likely to select for antigenic novelty. Indeed, even critics of the evolvability-as-adaptation hypothesis agree that plausible examples of natural selection that promote evolvability are most likely to be found in antigenic variation loci of microbial pathogens [1]. To date, however, empirical evidence of natural selection acting to promote evolvability is primarily correlative and indirect [6], [15]. Here we provide direct evidence of natural selection acting to promote antigenic evolvability in a well characterized microbial pathogen system.

**Figure 1:**
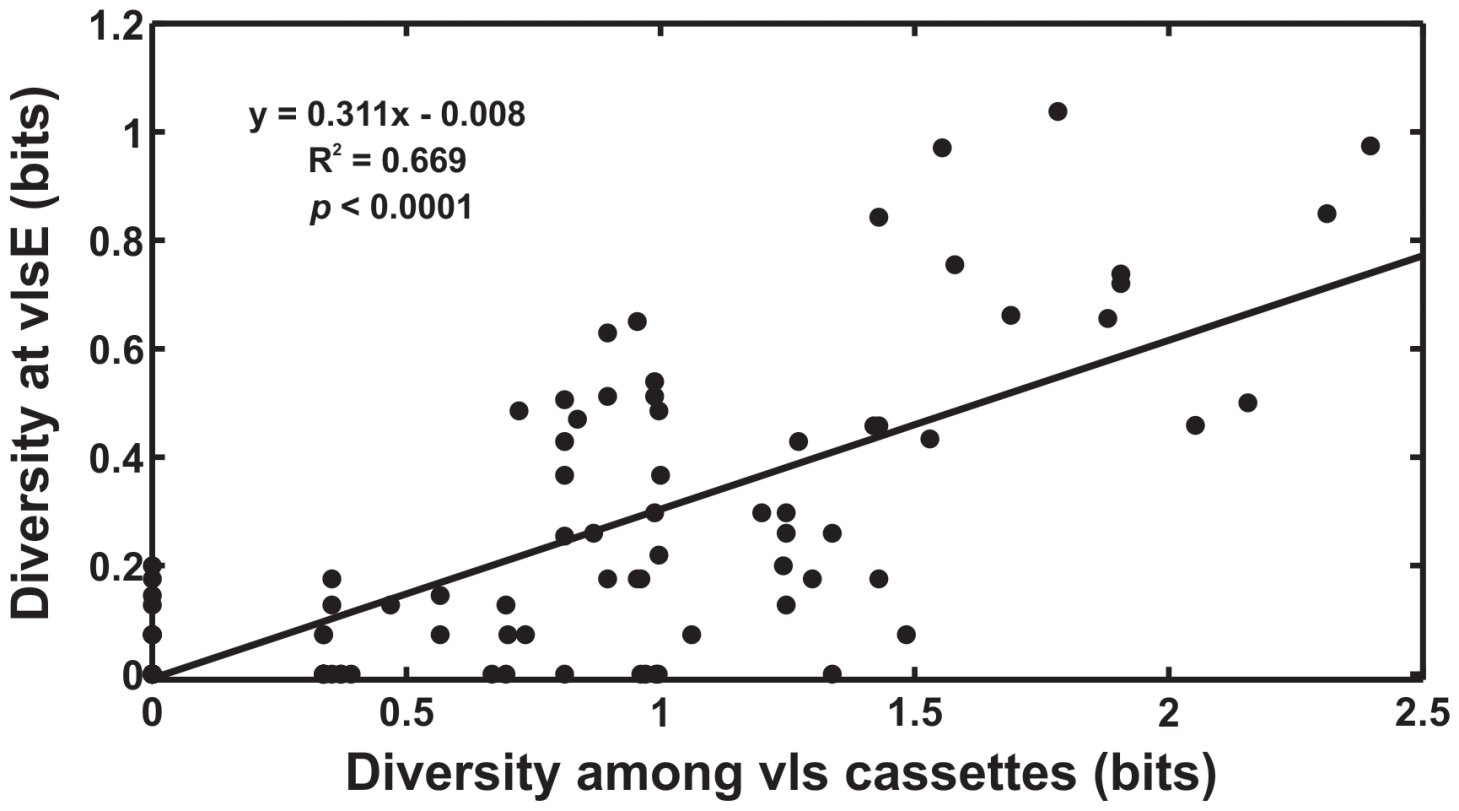
Evolvability of *vlsE* is tightly correlated with sequence diversity among the unexpressed *vls* cassettes. The level of sequence diversity at individual sites among the unexpressed *vls* cassettes was tightly correlated with the rate of sequence change at the corresponding sites in *vlsE* during experimental infections (R^2^ = 0.67, F(1,200) = 403.6, p<0.0001) (data from [21]). Thus, increasing diversity among the unexpressed cassettes will result in a corresponding increase in the antigenic evolvability at VlsE. Sequence diversity among the unexpressed cassettes was calculated as the entropy (bits) at each site in the 15 unexpressed cassette sequences; the rate of sequence change in the *vlsE* variants during experimental infections in mice was calculated as the entropy (bits) at each site of the 113 expressed antigen sequences. The diversity at *vlsE* in these data, from immunodeficient mice, results primarily from mutational inputs from the *vls* cassettes.

During vertebrate host infections, the immune system repeatedly eliminates lineages expressing antigens that are not sufficiently different from those previously expressed in the host. Lineages with greater potential to produce novel antigens – lineages with greater antigenic evolvability – are likely to be favored by natural selection due to their ability to rapidly adapt to the immune response of the host. In the Lyme disease bacterium, *Borrelia burgdorferi*, rapid evolution of the surface antigen, VlsE, is required for immune evasion and long-term infection in vertebrate hosts [16], [17], [18], [19], [20]. *In vivo* studies have shown that the *vlsE* antigen expression locus is indeed highly evolvable; a near-complete replacement of *vlsE* alleles occurs every 14–28 days in experimentally infected mice [21]. Novel VlsE antigens are generated through unidirectional recombination of a segment of one of the several unexpressed, paralogous *vls* cassettes into the *vlsE* expression site; six regions in the unexpressed *vls* cassettes are known to vary among cassettes and correspond to the antigenically important extracellular loop structures of the VlsE protein [18], [22], [23]. Previous studies have shown that novel VlsE antigens produced by recombination between *vls* cassettes and *vlsE* are not recognized by antibodies that target previously detected VlsE antigens [24]. Importantly, the evolvability of the *vlsE* locus during infection is tightly correlated with the amount of diversity among the unexpressed *vls* cassettes, as mutations in the *vlsE* locus are rare except by recombination (Fig. 1) [21]. Natural selection could therefore promote evolvability of the VlsE antigen by favoring lineages with greater genetic diversity among the *vls* unexpressed cassettes.

The evolutionary history of the *vls* antigenic variation system in *B. burgdorferi* is experimentally tractable as the reservoir of unexpressed cassettes maintains an historical record of past natural selection. Additionally, the reading frame in the unexpressed cassettes and the *vlsE* expression site is conserved making it possible to predict the amino acid sequence that would result due to recombination into *vlsE*. The proportion of ‘synonymous’ and ‘non-synonymous’ differences in the predicted reading frame of the unexpressed cassettes can be used to test whether natural selection preferentially favors among-cassette diversity that would alter the amino acid sequence of VlsE after recombination. We compared synonymous and non-synonymous differences among unexpressed cassettes within each of twelve *B. burgdorferi* strains [25] to statistically test the hypothesis that natural selection favors genetic diversity among the *vls* unexpressed cassettes in order to promote antigenic evolvability. We also examined evolutionary sequence changes in the unexpressed cassettes during experimental infections of laboratory animals. We use these findings to address the hypothesis that natural selection promotes antigenic evolvability and propose a model for the evolution and evolvability of antigenic variation systems of microbial pathogens.

## Results

### Diversifying selection in *vls* unexpressed cassettes indicates selection favoring antigenic evolvability at VlsE

**Figure 2:**
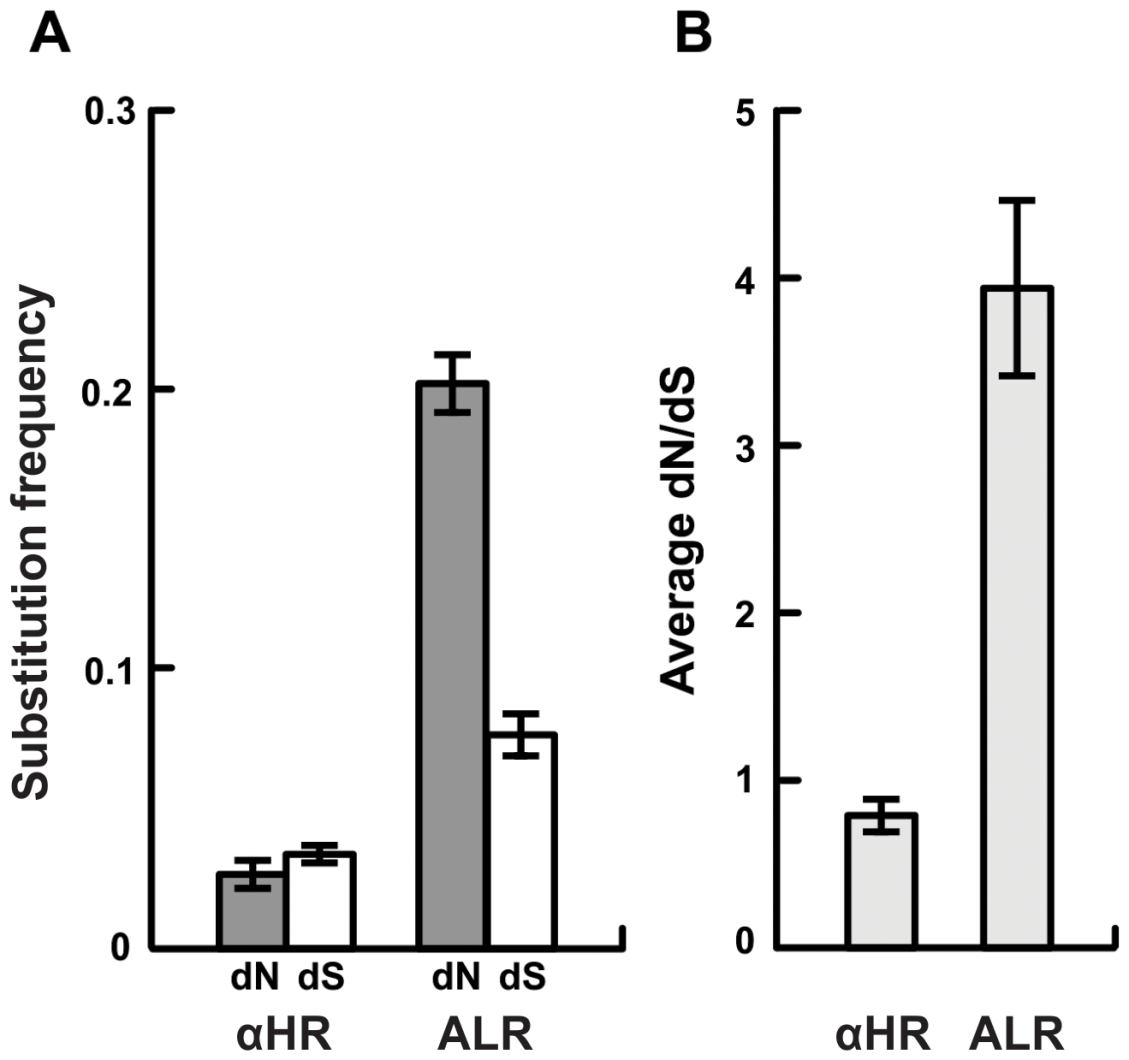
Strong selection for amino acid diversity among the *vls* unexpressed cassettes. **A**) The frequency of non-synonymous differences per non-synonymous site (dN) is significantly greater than the frequency of synonymous differences per synonymous site (dS) in the regions of the cassettes that correspond to the antigenic loop domains (ALR) of each strain suggesting strong positive selection. In contrast, synonymous and non-synonymous differences occur at similar frequencies in the regions of the unexpressed cassettes that correspond to the conserved alpha helical domains (αHR). **B**) The ratio of non-synonymous to synonymous polymorphisms (dN/dS) in the unexpressed cassettes of each strain is far greater than one in antigenic loop regions (ALR), suggesting diversifying selection, and slightly less than one in alpha helical regions, suggesting purifying selection or neutral evolution. Values are reported as the mean of all pair-wise comparisons of unexpressed cassettes within each strain averaged over all 12 strains (± standard error). Summaries of the statistical tests of diversifying selection are provided in Table S2.

**Figure 3:**
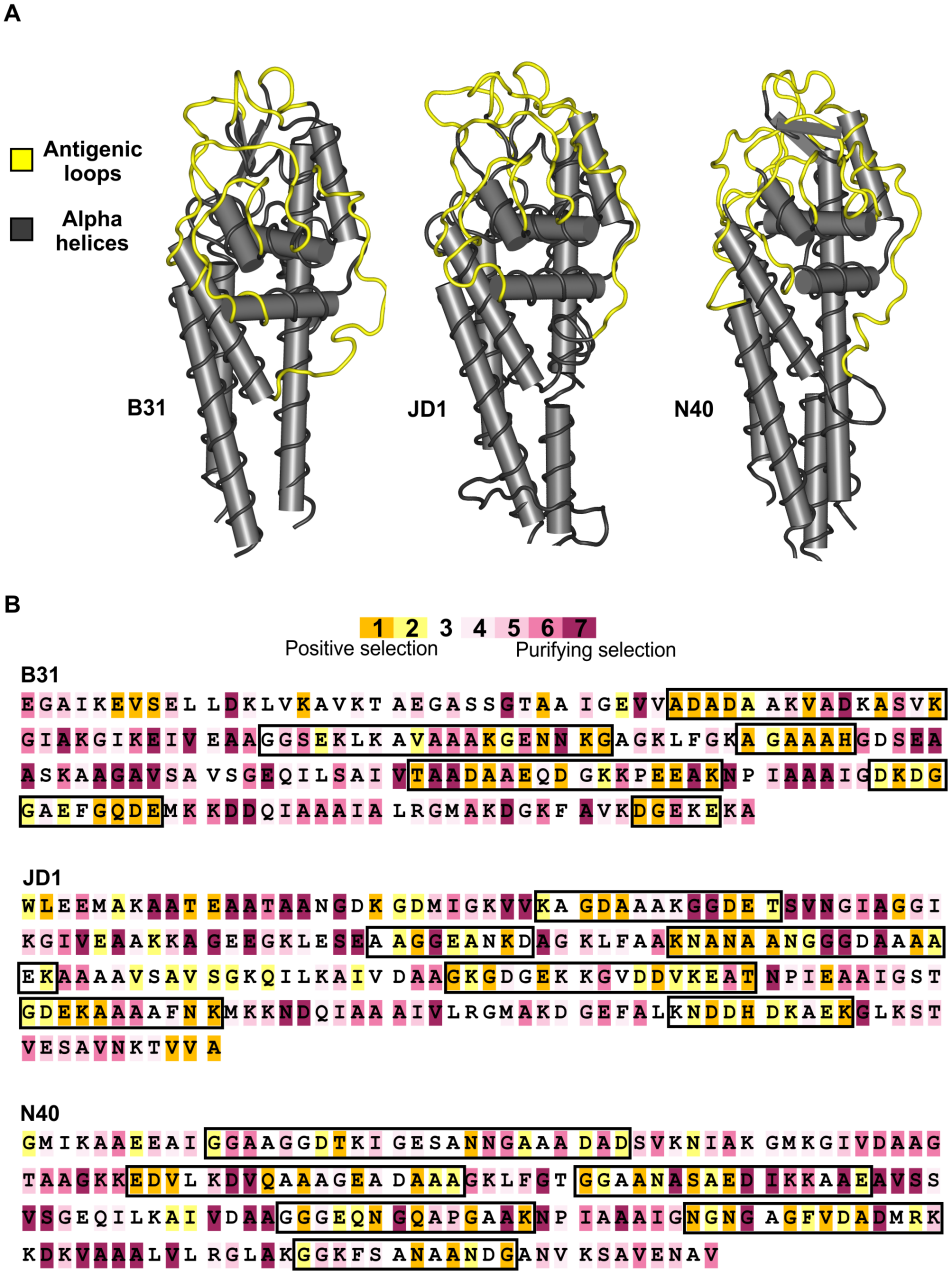
Strong diversifying selection is localized to the antigenically important regions of the unexpressed cassettes. **A**) The six variable regions of the unexpressed cassettes are expressed as loop structures (yellow) exposed on the surface of the bacterium in all *B. burgdorferi* strains examined. The conserved alpha helical regions (grey) encode structural alpha helices in the expressed protein. Structural models of the expressed VlsE protein are shown for three evolutionarily divergent strains of *B. burgdorferi*: B31 (crystal structure reported by Eicken et al. 2002), JD1 (predicted structure), and N40 (predicted structure) **B**) Codon-by-codon analyses of the unexpressed cassettes identified a high frequency of positively selected codons (highlighted in orange) in the regions that correspond to the antigenically-important loop domains (demarcated by black boxes). The majority of codons in the regions that correspond to the alpha helical domains are under stabilizing selection (highlighted in purple). One translated cassette sequence from each of the three evolutionarily divergent strains is shown. There is strong statistical support for positive selection in 28–43% of the codons in regions of the cassettes homologous to the antigenic loop domains of *vlsE* compared to only 0–5% in regions homologous to the alpha helical domains of *vlsE*.

Diversity among the unexpressed *vls* cassettes is correlated with the evolvability of the *vlsE* expression locus (Fig. 1). This relationship results from the fact that nearly all of the sequence evolution at *vlsE* is generated through unidirectional recombination of a segment of the unexpressed *vls* cassettes into *vlsE* [21]. Thus, evidence of selection for increased diversity among *vls* cassettes would also be evidence that natural selection favors elevated antigenic evolvability at the *vlsE* expression locus. To test the hypothesis that natural selection has favored increased antigenic evolvablity of VlsE in *B. burgdorferi*, we analysed the unexpressed *vls* cassette sequences in 12 independent strains of the bacterium for signatures of intragenomic diversifying selection. Such diversifying selection was strongly supported by three lines of evidence. First, non-synonymous differences (per non-synonymous site) are substantially more frequent than synonymous differences (per synonymous site) among the six regions of the unexpressed *vls* cassettes that correspond to the antigenically important loop regions of VlsE (Fig. 2A). This pattern was observed in all 12 strains analyzed and was statistically significant in 10 strains despite inclusion of data from the highly conserved regions of the cassettes that correspond to the alpha helical domains of VlsE in the statistical analyses (see Table S2). In contrast, synonymous differences were more common than non-synonymous differences in the regions of the unexpressed cassettes homologous to the sequences encoding alpha helices on the expressed protein (Fig. 2B). Taken together, these observations indicate that selection favors the potential for amino acid diversity at regions of the unexpressed cassettes that encode antigenic epitopes upon recombination into *vlsE* whereas regions of the unexpressed cassettes that are homologous to the alpha helices of VlsE are evolving under neutral or purifying selection. Second, and consistent with these results, codon-by-codon inferences which use a Bayesian posterior distribution to assign confidence to the ratio of non-synonymous and synonymous substitutions at each codon identified a large proportion of amino acid residues under positive selection in regions of the cassettes that correspond to the antigenically important loop domains on the surface of VlsE; most residues in the cassettes that correspond to the alpha helical domains in VlsE show signatures of stabilizing selection (Fig. 3). Finally, a codon substitution model allowing for heterogeneous selective pressures among sites in the cassettes was significantly more likely in all strains when sites under positive selection were permitted in the model [26] compared to a model allowing only purifying selection and neutral evolution [27](Likelihood ratio test, p<0.001). The evidence of selection for diversity among the *vls* cassettes provides evidence of selection for elevated antigenic evolvability at VlsE.

**Figure 4:**
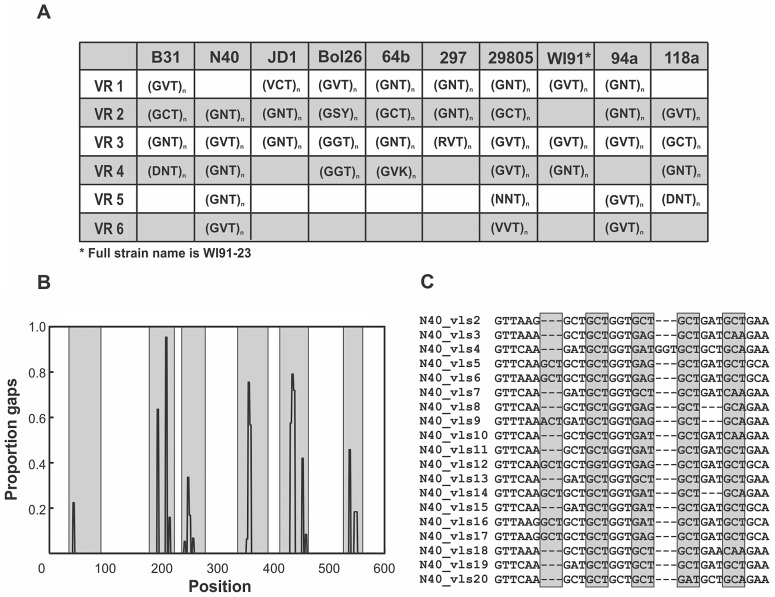
Mutation-prone tandem-repeat motifs are conserved in regions of the unexpressed cassettes that correspond to the antigenic loop domains. **A**) Highly-mutable tandem-repeat motifs were identified in the majority of the regions of the cassettes that correspond to the antigenically important loop domains in each strain despite little sequence conservation among strains. Polymorphic sites, denoted with ambiguities following the IUPAC standard, occur primarily in the second codon position. Although the tandem-repeat motifs are similar within the *vls* unexpressed cassettes of each strain, the number of repeats varies due to insertion and deletion (indel) mutations. **B**) Tandem-repeat motifs are associated with high frequencies of indel mutations in antigenic loop regions (shaded). The association between tandem repeats and indel mutations in the regions corresponding to the antigenic loop domains is represented as the frequency of sites with gaps in the nucleotide alignment of unexpressed cassettes in strain N40. Indels are present at significantly lower frequencies in antigenic loop regions of the unexpressed cassettes that do not contain tandem repeats (Fig. S1) and are absent in the conserved alpha helical regions that are devoid of tandem-repeat motifs. **C**) An alignment of antigenic loop region 2 of the N40 unexpressed cassettes illustrates the 3 bp tandem-repeat motifs common to antigenic loop regions of all strains.

Diversity among the cassettes is further increased by the presence in all strains of highly-mutable tri-nucleotide tandem-repeat motifs in regions homologous to the antigenically important loop structures on VlsE (Fig. 4A). These repeats are associated with a high frequency of insertion-deletion (indel) mutations (Fig. 4B, Fig. S6) that occur as triplets in-line with the reading frame and therefore do not result in frameshift or nonsense mutations when recombined into the *vlsE* expression locus. Length variation due to tandem repeats is a common source of sequence diversity in *vls* unexpressed cassettes of *B. burgdorferi* (Fig. 4C). In fact, indel events were significantly more likely in antigenic loop regions that contained tandem repeats compared to those in which no repeats were detected (Permutation test, p<0.02) (Fig. S1). Interestingly, tandem repeat structures are maintained in the unexpressed cassettes of all strains despite an almost complete absence of sequence identity between strains, making them one of the only conserved features among the antigenically important domains of the *vls* unexpressed cassettes (Fig. 4A). The tri-nucleotide repeats are highly conserved at the first and third codon positions but are significantly more variable at second codon positions resulting in differences in the amino acids they encode (Kruskal-Wallis test, p<0.0001, Fig. 4C, Fig. S2). Thus, expansion and contraction of the tri-nucleotide tandem-repeat motifs results in length variation in regions of the cassettes homologous to the antigenic loop domains of VlsE but does not produce tracts consisting of a single amino acid residue.

### Diversifying selection is detectable despite concerted evolution among cassettes

The unexpressed *vls* cassettes, like all repeated sequences, are susceptible to homogenization through gene conversions, duplications, and deletions [28], [29], [30], [31], [32]. This is supported by a strong phylogenetic signature of concerted evolution in the cassettes – a pattern of diversity in which sequence divergence among the cassettes is far greater between strains than within strains (Fig. S3). Indeed, amino acid sequence divergence among the unexpressed cassettes within strains is very low at regions homologous to the alpha helical domains of VlsE (2–8%) and moderate (26–48%) in regions homologous to the surface-exposed antigenic loops (Fig. S3B, diagonal). By contrast, cassettes from different strains are very divergent at both alpha-helical regions (27–42% amino acid divergence; Fig. S3B, below diagonal) and the surface-exposed antigenic loop regions (71–88%; Fig. S3B, above diagonal). Although recombination from the cassettes into the *vlsE* expression locus is unidirectional and does not affect the sequence of unexpressed cassettes [33], gene conversion between cassettes will homogenize the sequences and could explain the observed pattern of concerted evolution. The signal of diversifying selection that we detect among the cassettes within strains is all the more remarkable given the clear tendency for gene conversion to eliminate differences among the cassettes and thus eliminate evidence of past natural selection for increased diversity.

### The mutation rate in unexpressed cassettes is much greater than the mutation rate at other loci

Sequence alterations in the *vls* unexpressed cassettes are much more common during the course of infections than at other genetic loci. The *vls* unexpressed cassettes of three clonal isolates sequenced after one year in experimentally infected mice had diverged from the inoculating strain by a total of 23 substitutions and 2 large deletions (Fig. S4, Fig. S5). In contrast, no mutations were observed at the *ospA* or IGS loci (this study), nor the *ospC* or *erp* loci [34], [35] of the same isolates (Table S3). These and other surface exposed proteins are not expected to experience diversifying selection as they are either not expressed during vertebrate infections or are down-regulated once antibodies against them are developed by the host [36], [37], [38], [39]. The majority of the substitutions introduced in the cassettes were identical to homologous sites from a different cassette and are likely to have resulted from gene conversion events between cassettes. Two single-nucleotide substitutions occurred in the unexpressed cassettes during experimental evolution that were not attributable to gene conversion: one in a region homologous to the antigenically important loop structures in VlsE and one in a conserved alpha helical region (Fig. S4). Interestingly, the substitution that occurred in an antigenically important region (cassette 2 of derived isolate 1) was non-synonymous, whereas the substitution that occurred in an alpha helical region (cassette 15 of derived isolate 3) was synonymous. These data are consistent with our analyses supporting diversifying selection on amino acid composition in the unexpressed cassettes.

## Discussion

Many pathogens rely on continual genetic changes to their antigens to rapidly adapt to the immune response and persist in their hosts. In *B. burgdorferi*, the rate of genetic change, or antigenic evolvability, at VlsE is tightly correlated with the amount of genetic diversity contained in the unexpressed *vls* cassettes (Fig. 1) [21]. Antigenic evolvability at VlsE is required for long-term infection in vertebrate hosts [16], [17], [18], [19], [20]. Previous studies have demonstrated that novel VlsE antigens, generated through recombination between *vls* cassettes and the *vlsE* expression site, are favored by natural selection because they are not recognized by antibodies that target previously detected VlsE antigens [21], [24]. Thus, *B. burgdorferi* lineages with greater diversity among the *vls* cassettes will have a selective advantage as they will be more antigenically evolvable (better able to repeatedly generate novel antigens) and thus be more likely to persist within hosts [19], [21]. The hypothesis that natural selection favors diversity among the *vls* cassettes and promotes antigenic evolvability was supported by molecular evolutionary analyses of the cassettes of 12 *B. burgdorferi* strains (Fig. 2, Fig. 3). Polymorphisms among the unexpressed cassettes that would result in non-synonymous changes in the antigenic loops of VlsE were up to eight times more frequent than expected by random mutation alone (Table S2), and numerous codons showed strong signatures of diversifying selection (Fig. 3B). In addition, mutation-prone tandem repeats were conserved in all strains despite a near complete sequence divergence among the *vls* cassettes (Fig. 4A). Conservation of these mutation-prone structures is consistent with the hypothesis that natural selection acts to maintain mutable sequence structures that promote diversity among unexpressed cassettes. Diversification among *vls* cassettes promoted by natural selection and mutable repeat structures is detectable despite the tendency for gene conversion to eliminate differences among the cassettes and thus eliminate evidence of past natural selection for increased diversity. These results support the hypothesis that natural selection favors those mutations that increase the diversity among the unexpressed cassettes in order to promote antigenic evolvability in a primary surface antigen of *B. burgdorferi*.

Despite the limited sequence identity at the *vls* unexpressed cassettes among strains, six clearly identifiable regions of high variability were maintained in all strains. When recombined into the *vlsE* expression locus, these regions are expressed as antigenically important loop structures on the surface of the bacterium [21], [23](Fig. 3A). Our analyses revealed that diversity among the unexpressed cassettes is elevated by natural selection favoring mutations that code for amino acid changes in the antigenically important regions (Fig. 2, Fig. 3), thus elevating antigenic evolvability at VlsE. The regions of the unexpressed cassettes that correspond to antigenically important loop regions contained significantly more non-synonymous polymorphisms than synonymous polymorphisms, supporting the hypothesis that variation in the cassettes is maintained by diversifying selection. This conclusion was supported by three independent statistical tests of diversifying selection on the cassettes. Importantly, these signatures of selection were strong enough to overcome acknowledged detection limitations resulting from averaging frequencies of non-synonymous polymorphisms over both antigenic loop and conserved alpha helical regions, using samples from a single species [40], and using analytical methods which yield conservative estimates [41]. The high rate of non-synonymous polymorphisms in the unexpressed cassettes likely results from random mutations that are favored by natural selection if they enhance antigenic evolvability at VlsE, as no mutational mechanism that is biased toward amino acid substitutions has been described.

Antigenic evolvability at VlsE is also elevated by insertion-deletion (indel) mutations at unstable tandem-repeat motifs, which are present in all *B. burgdorferi* lineages analyzed. These repeats promote diversity in regions of the unexpressed cassettes that correspond to the antigenic loop domains in VlsE (Fig. 4, Fig. S1). Tandem repeats are prone to length mutations caused by slipped-strand mispairing during DNA replication [9], [42], accounting for the high frequency of indel mutations observed in these regions (Fig. 4B). Tandem repeats have previously been reported in and around antigens of numerous pathogens where the resulting increase in mutation rate is hypothesized to be an adaptation to facilitate rapid adaptation to the host immune response [6], [43]. Their presence in the unexpressed *vls* cassettes of *B. burgdorferi* coincides with strong signatures of diversifying selection among the cassettes and provides additional empirical evidence for the adaptive significance of tandem repeats in pathogens.

All repeats observed in *B. burgdorferi* occur as triplets in line with the reading frame and thus have the potential to alter antigenic epitopes, when recombined into VlsE, without introducing stop codons or frameshifts that would have deleterious effects on the protein structure. The tri-nucleotide repeats show little variation at the first and third codon positions but are significantly more variable at second codon positions resulting in differences in the amino acids they encode (Fig. 4A, Fig. S2). Thus, expansion and contraction of the tri-nucleotide tandem-repeat motifs results in length variation in antigenic loop regions of the cassettes but do not produce tracts consisting of a single amino acid residue. Further experimental data are needed to establish that tandem repeats in the unexpressed cassettes are maintained by natural selection in evolving populations. Nevertheless, the presence of the highly-mutable tandem repeat motifs in all strains despite the absence of sequence homology (Fig. S3B) suggests that mutable sequences may be selectively maintained as a mechanism to generate the genetic diversity among the cassettes that is needed to elevate antigenic evolvability at VlsE. This explanation is consistent with the analyses supporting diversifying selection in the unexpressed cassettes.

Ascertaining whether evolvability is a byproduct of selection on other phenotypes or is, itself, the object of natural selection presents an empirical challenge, especially in natural populations in which the consequences of putative evolvability differences cannot be tested directly [1], [2], [5], [7], [8], [43]. The antigenic variation system of *B. burgdorferi* provides a *measurable* phenotype, however, that can be used to test whether evolvability has been the object of natural selection. The amino acid diversity among the unexpressed *vls* cassettes determines the rate of evolutionary change, or evolvability, at *vlsE* (Fig. 1). Thus, the population genetic analyses described above provide clear evidence of selection in favor of amino acid diversity at the *vls* cassettes that enhances evolvability at VlsE. It is unlikely that among-cassette diversity generated by point mutations or indels is directly favored by natural selection or is favored as a byproduct of a function unrelated to the evolvability of the *vlsE* expression locus, because the cassettes are not expressed and serve no known function aside from recombination with *vlsE*. Rather, *B. burgdorferi* lineages with greater among-cassette diversity are more likely to persist evolutionarily because of their increased capacity for rapid sequence evolution, or evolvability, at *vlsE*.

There are two potential scenarios that could account for our observation that natural selection promotes increased antigenic evolvability in *B. burgdorferi*. Selection could favor populations that can rapidly generate novel VlsE antigens during an infection because this enables rapid adaption to changes in the immune response and persistence within a host [19], [20], [24]. Alternatively, selection could favor individual cells which produce offspring that tend to be antigenically different because this increases the likelihood that offspring will survive the host immune response. These two possibilities—population-level and individual-level—are nearly indistinguishable in pathogens like *B. burgdorferi* because infections are likely to be derived from a small number of highly related cells, which has the effect of aligning the fitness interests of individuals and populations [10], [11]. In either scenario, moreover, more diverse sets of cassettes are expected to prevail over less diverse cassettes via the well-understood population genetic process of hitchhiking, rising to high frequency as a consequence of their association with VlsE antigens that escape immune surveillance.

Sexual eukaryotic pathogens conceivably experience fluctuating selective pressures similar to those experienced by *B. burgdorferi* and might be expected to exhibit signatures of selection on evolvability similar to those we have described here. In sexual populations, however, recombination will tend to separate the genetic drivers of rapid sequence evolution (analogous to *vls* cassette diversity in *B. burgdorferi*) from the beneficial alleles they create (VlsE escape antigens), thereby inhibiting hitchhiking [1], [44]. For this reason, the evolution of unambiguous signatures of selection on evolvability such as those we have reported here is likely to be restricted to cases of tight genetic linkage.

The data reported here exhibit the genetic signatures expected given selection on antigenic evolvability. Future experimental evolution assays can be used to experimentally validate these conclusions and to further dissect the molecular mechanisms involved. For example, assays competing isogenic *B. burgdorferi* strains that differ only in the diversity among *vls* cassettes in a repeated mouse-tick-mouse transmission cycle would provide an experimentally-controlled evaluation of the strength of selection on evolvability in this system. Analyses of the mutations introduced into the cassettes of each strain at each mouse-tick-mouse transmission could also elucidate the role of tandem repeats and other mechanisms that result in greater diversity among cassettes. In particular, comparing experimental infections in immunologically active and immunocompromised mice can determine the role of cassette diversity in establishing and maintaining persistent infection during an adaptive host immune response.

Our results support a model of evolution in the *vls* unexpressed cassettes in which strong diversifying selection leads to elevated amino acid diversity in regions that correspond to antigenically important domains in order to promote the evolvability at VlsE that allows for continual immune evasion. Such selection for cassette diversity could be a common strategy for maintaining antigenic evolvability in a diverse range of pathogens that generate antigenic variation by intragenomic recombination [45], [46], [47], [48], [49], [50], [51]. For example, polymorphisms in the semi-variable and hyper-variable regions of the unexpressed cassettes of the *pilE* locus of *Neisseria gonorrhoeae* are maintained despite common gene conversion events [49], possibly due to evolutionary processes similar to those described here. Similar analyses to those conducted in this study can be used to establish whether the model of cassette evolution proposed here maintains antigenic evolvability in other pathogens. Further, the finding that selection promotes antigenic evolvability in microparasites may offer an explanation for numerous observations of sophisticated variation systems used to adapt to rapidly changing environments [43], [45], [46], [47].

## Materials and Methods

### Diversifying selection in *vls* unexpressed cassettes

We analyzed the genetic diversity of the *vls* unexpressed cassettes both within and among *B. burgdorferi* strains for which genome sequence data was available [25] (Table S1). Amino acid sequences of individual cassettes from all strains were aligned using MAFFT [52] and converted into the corresponding codon alignment using PAL2NAL [53].

The hypothesis that diversity among unexpressed cassettes is favored by natural selection to promote antigenic evolvability was tested using three molecular evolutionary analyses. First, the proportion of non-synonymous polymorphic nucleotides (dN) and synonymous polymorphic nucleotides (dS) were estimated [54] for all pair-wise comparisons among unexpressed cassette alignments within each strain. The average across the pair-wise comparisons for dN and dS were calculated for both antigenic loop and alpha helical regions to test for evidence of selection. Evidence of selection was detected using a Z-test of the hypothesis that dN is significantly different than dS across the complete alignment (antigenic loop and alpha helical regions) in each strain [55] using the MEGA v. 5.5 software [56] with variance estimates calculated using 1000 bootstrap replicates. Second, codon-by-codon analyses of positive selection among cassettes within each strain was conducted using the Selecton v. 2.4 server [41]. Codons under positive or purifying selection were identified based on the 95% confidence interval of Bayesian posterior dN/dS estimates at each codon in the alignment. The hypothesis of positive selection was further tested via likelihood ratio tests comparing of the likelihood of the M8a model of evolution which allows only stabilizing selection and neutral evolution [26] to the likelihood of the M8 model which also allows positive selection [27].

Tandem-repeat motifs were identified using the mreps server [57] and majority-rule consensus sequences of the repeats were reported (75% threshold). The frequency of insertion-deletion (indel) mutations was calculated for each strain as the proportion of nucleotide sequences containing gaps at each site in the multiple sequence alignments of the cassette regions (excluding large indel mutations that are not the result of tandem repeat length variation).

Structural models of VlsE in *B. burgdorferi* strains JD1 and N40 were predicted using the SWISS-MODEL server [58] based on homology to the 1L8W chain A of the resolved protein structure in strain B31 [23]. Structural models for VlsE in *B. burgdorferi* strain JD1 were determined using the reported VlsE sequence (Genbank [CP002306]), whereas the protein structure in strain N40 was predicted by replacing the cassette region of *vlsE* in B31 with the unexpressed cassettes of N40.

A nucleotide Neighbour-Joining (NJ) phylogeny (Jukes Cantor distance, 1000 bootstrap replicates) was constructed in Geneious v. 5.3 [59]. Pair-wise amino acid distance matrices were produced by averaging the Hamming distances of nucleotide sequence alignments within and among strains at both the antigenic loop and alpha helical regions.

### Experimental evolution

A clonal isolate of *B. burgdorferi* strain N40 [60] was intradermally inoculated into three C3H/HeN mice and re-isolated after 12 months from the blood (derived isolate 1(36B), derived isolate 2(44B), and derived isolate 3(39B)) as previously described [34]. The clonal N40 parent isolate (cN40) and each of the derived isolates were grown from frozen stocks at 34°C in BSK-H medium supplemented with 6% rabbit serum (Sigma Aldrich) to a density of ∼5*10^7^ cells/ml and the genomic DNA was purified using the DNeasy Blood and Tissue Kit protocol for gram negative bacteria (Qiagen; Valencia, CA). The *vls* cassette region from each isolate was cloned into BigEasy v2.0 Linear Cloning Kit (Lucigen; Middleton, WI) by either 1. ligating total genomic DNA treated with Mung Bean Nuclease and DraI (New England Biolabs (NEB); Beverly, MA) into the cloning vector (derived isolate 2(44B)) or 2. ligating a long-range PCR fragment containing the unexpressed cassettes into the cloning vector (isolates cN40, 36B, and 39B). Long-range PCR amplification was conducted using the primers N40-vlsLR-R (5′ Phos - GCT GGA CTT GAA TTT GGT AGG GAT TC 3′) and N40-vlsLR-F (5′ Phos - GGT GAT GGT GCC GAT TCA AAA TCT GG 3′) which anneal to the unique conserved regions flanking the unexpressed cassettes. The PCR reactions contained 2–6 ng/µl of genomic DNA, 0.2 mM of each dNTP, 0.4 µM of each primer, 7% DMSO, 1× GC buffer and 0.02 U/µl of Phusion Hot Start II DNA Polymerase (NEB) and were amplified with 25 cycles of 30 s at 98°C and 90 s at 72°C.

BigEasy vectors containing an intact cassette region were amplified in *E.coli* and isolated using a Qiagen Mini-prep kit. The TSA cell line used in transformation of the BigEasy vector (Lucigen) contains *RecA* and *EndA* mutations that make them recombination-deficient and minimize the chance that gene-conversion was introduced during cloning. Purified plasmid DNA was sheared using a Nebulizer (Invitrogen; Carlsbad, CA) to 500–3000 bps, purified by ethanol precipitation, and subcloned using the Zero Blunt PCR cloning kit for sequencing (Invitrogen). Additionally, the *ospA* and *rrs-rrlA IGS* loci were PCR amplified from each culture as previously described [61] and sequenced for comparison. Each shotgun sequencing fragment was aligned independently to the sequence reported for N40 plasmid lp36-1 [25] (Genbank [CP002230]) to minimize errors in shotgun sequencing assembly. All regions with reported sequence changes received between 6× and 11× coverage in the assembly. Additional notes on the cassette sequencing methodology are provided with supplemental Figure S3.

## Supporting Information

Figure S1 **Indels are more abundant in antigenic loop regions containing 3 bp tandem repeat motifs.** The average number of indel events in regions of the cassettes corresponding to antigenic loop regions (ALR) of VlsE was significantly larger in those that contained tandem repeats compared to those in which no repeats were detected (Permutation test, p<0.02). This suggests that tandem repeats contribute to diversification among the cassettes by increasing the rate of indel mutations and contributing to length variation among cassettes in the antigenic loop regions.
(TIF)

Figure S2 **Greater diversity at second codon position of tandem repeats.** Diversity at the second positions of identified 3 bp tandem repeat motifs, which are inline with the reading frame, were significantly greater than diversity at the first and third positions (Kruskal Wallis test, p<0.0001). All differences at the second positions resulted in amino acid changes in the expressed proteins. Diversity was measured by parsing each of the 3 bp elements within a repetitive region and calculating the entropy at the first, second, and third position.
(TIF)

Figure S3 **Concerted evolution of the** ***vls*** **unexpressed cassettes.** **A**) The unrooted Neighbor-Joining phylogeny of individual unexpressed cassettes demonstrates high degrees of similarity within *B. burgdorferi* strains and high degrees of divergence among cassettes of different strains. This pattern of concerted evolution is typical of multi-copy sequences and results from sequence homogenization within strains due to gene conversion. The nucleotide Neighbour-Joining (NJ) phylogeny (Jukes Cantor distance, 1000 bootstrap replicates) was constructed in Geneious v. 5.3 [59]. Of the eight highly-supported evolutionary lineages of unexpressed cassettes, five consist of unexpressed cassettes from a single strain and the remaining three contain sequences from two or three strains. **B**) Diversity among the *vls* unexpressed cassettes within each strain is localized to six antigenic loop regions. In both antigenic loop and alpha helical regions, cassettes are far more divergent between strains compared to diversity contained among cassettes within individual strains. Although recombination from the cassettes into the *vlsE* expression locus is unidirectional and does not affect the sequence of unexpressed cassettes [18], gene conversion between cassettes will homogenize the sequences and could explain the observed pattern of concerted evolution. Homogenization of cassettes within strains reduces the potential for antigenic variation and thus strengthens the selective pressure to produce the genetic diversity necessary for antigenic evolvability at VlsE. Pair-wise amino acid distance matrices were produced by averaging the Hamming distances of nucleotide sequence alignments within and among strains at both the antigenic loop and alpha helical regions. The genetic distances shown are averaged across pair-wise comparisons of amino acid sequences from antigenic loop regions (above diagonal) and alpha helical regions (below diagonal). Values along the diagonal represent the average genetic distance between cassettes within each strain. **C**) Moderate sequence divergence was observed between cassettes belonging to strains that co-occurred on the same branch of the phylogeny. Three evolutionary lineages in the phylogenetic analyses consisted of unexpressed cassettes from more than one strain. The average pair-wise genetic distance among strains at alpha helical regions (below diagonal) and antigenic loop regions (above diagonal) shows a slight increase in diversity between strains compared to divergence within strains (along diagonal) indicating incipient divergence. Divergence among strains was not sufficient to produce a signal in the phylogenetic analysis, possibly due to recent ancestry, which is further supported by the observation that strains with similar unexpressed cassettes contain the *vls* antigenic variation system on the same linear plasmid (Table S1).
(TIF)

Figure S4 **Experimental evolution of** ***B. burgdorferi*** **reveals mutations caused by gene conversions among unexpressed cassettes.** Evolutionary changes observed in the cassette regions during experimental infections are characterized primarily by gene-conversions and large deletions. The parental N40 isolate differs from a previously sequenced N40 laboratory isolate in a 70 bp segment of cassette 19 which contains 12 substitutions and a 6 bp deletion. These sequence changes are identical to the homologous region of cassette 20 and are shared in all derived strains. Mutations that occurred during infection consisted of gene conversion events in derived isolates 1 and 3 and large deletions in derived isolates 2 and 3. The mutations in cassette 13 of derived isolate 3 are depicted with different colors to represent gene conversion from different donor cassettes. At least 6 overlapping gene conversion events from other cassettes are necessary to account for the mutations that occurred in this cassette. Of the two single-nucleotide substitutions in cassettes occurred during experimental evolution, the substitution that occurred in an antigenically important region (cassette 2 of derived isolate 1) was non-synonymous, whereas the substitution that occurred in a alpha helical region (cassette 15 of derived isolate 3) was synonymous.
(TIF)

Figure S5 **Southern hybridization confirms large deletions in the** ***vls*** **cassette region.** The large deletions observed in the unexpressed cassette region of derived strains 2 and 3 (Fig. S4) were confirmed by Southern blot of genomic DNA isolated from the *B. burgdorferi* strains. Genomic DNA was purified from the parental strain and each of the three derived strains as described in the text and digested with DraI endonuclease (NEB) which cuts at the recognition site, TTTAAA. Recognition sites for this enzyme occur throughout the AT rich genome of *B. burgdorferi* but are present at only one site within the *vls* unexpressed cassette region of each strain. The digested DNA was separated for four hours on a 0.4% agarose gel, transferred to a nylon membrane, and allowed to hybridize with two fluorescein labeled probes specific to the *vls* cassette sequences (5′ *Fluor*- TTC GGT TRG TGG KGA GCA GA 3′ and 5′ *Fluor*- ACA ATC CCC TTC ATC CCC TTA 3′). Southern blot analysis revealed two fragments (∼3 kb and ∼4.5 kb) in both the parental strain (cN40) and derived isolate 1 (36B) consistent with the single DraI site expected 2949 bp from the beginning of cassette 1 of a 7.467 kb fragment given the sequence data obtained in this study. The single fragment (∼4.3 kb) apparent in derived isolate 3 (39B) is consistent with the single DraI site expected 4204 bp from the beginning of cassette 1 of a 4.381 kb fragment. The 177 bp fragment was likely not detected because it ran off the gel. Similarly, derived isolate 2 (44B) revealed only a single fragment (∼5.9 kb) which is consistent with the single expected DraI site 5893 bp from the beginning of cassette 1 in a 6.073 kb fragment.
(PDF)

Table S1 **Summaries of the silent cassette loci in** ***B. burgdorferi*** **strains analyzed in this study.**
(DOC)

Table S2 **Statistical analysis of positive selection within silent cassettes of** ***B. burgdorferi*** **strains.**
(DOC)

Table S3 **Genbank accession numbers of the** ***vls*** **cassette region,** ***ospA*** **and** ***IGS*** **loci sequenced in isolates after one year of experimental infection.**
(DOC)
