# Supplementary figures and images for: Natural Selection Promotes Antigenic Evolvability
Source: PLoS Pathog. 2013 Nov 14;9(11):e1003766. doi: 10.1371/journal.ppat.1003766 (PMC3828179; doi:10.1371/journal.ppat.1003766)

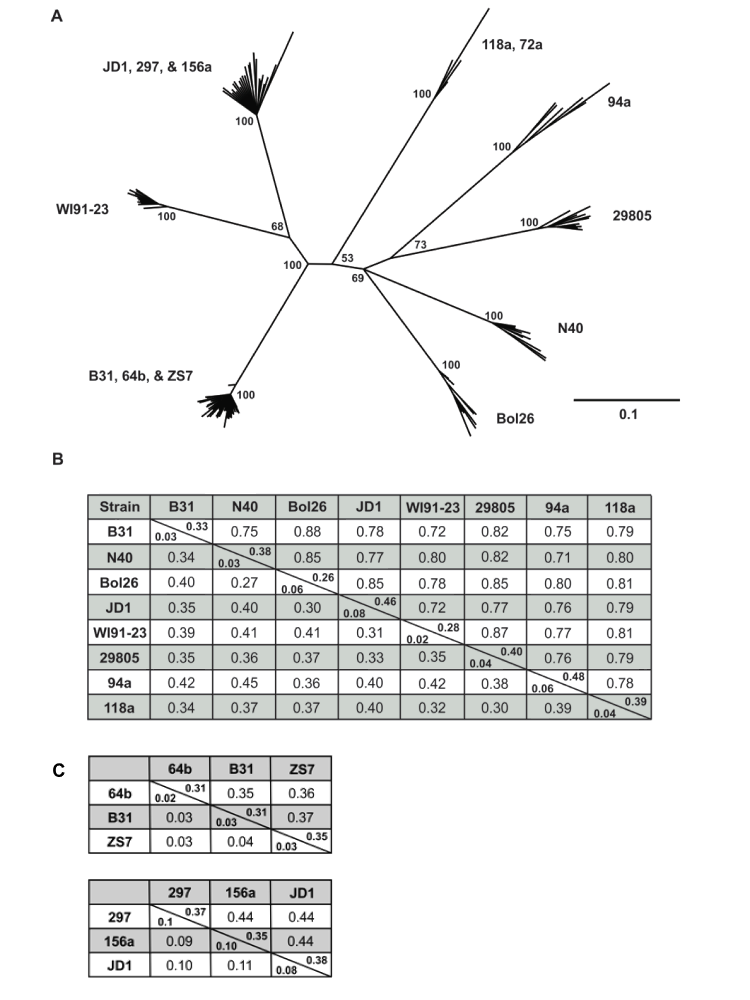

Supplement: Figure S3 — Concerted evolution of the vls unexpressed cassettes. A) The unrooted Neighbor-Joining phylogeny of individual unexpressed cassettes demonstrates high degrees of similarity within B. burgdorferi strains and high degrees of divergence among cassettes of different strains. This pattern of concerted evolution is typical of multi-copy sequences and results from sequence homogenization within strains due to gene conversion. The nucleotide Neighbour-Joining (NJ) phylogeny (Jukes Cantor distance, 1000 bootstrap replicates) was constructed in Geneious v. 5.3 [59]. Of the eight highly-supported evolutionary lineages of unexpressed cassettes, five consist of unexpressed cassettes from a single strain and the remaining three contain sequences from two or three strains. B) Diversity among the vls unexpressed cassettes within each strain is localized to six antigenic loop regions. In both antigenic loop and alpha helical regions, cassettes are far more divergent between strains compared to diversity contained among cassettes within individual strains. Although recombination from the cassettes into the vlsE expression locus is unidirectional and does not affect the sequence of unexpressed cassettes [18], gene conversion between cassettes will homogenize the sequences and could explain the observed pattern of concerted evolution. Homogenization of cassettes within strains reduces the potential for antigenic variation and thus strengthens the selective pressure to produce the genetic diversity necessary for antigenic evolvability at VlsE. Pair-wise amino acid distance matrices were produced by averaging the Hamming distances of nucleotide sequence alignments within and among strains at both the antigenic loop and alpha helical regions. The genetic distances shown are averaged across pair-wise comparisons of amino acid sequences from antigenic loop regions (above diagonal) and alpha helical regions (below diagonal). Values along the diagonal represent the average genetic distance be [file ppat.1003766.s003.tif]

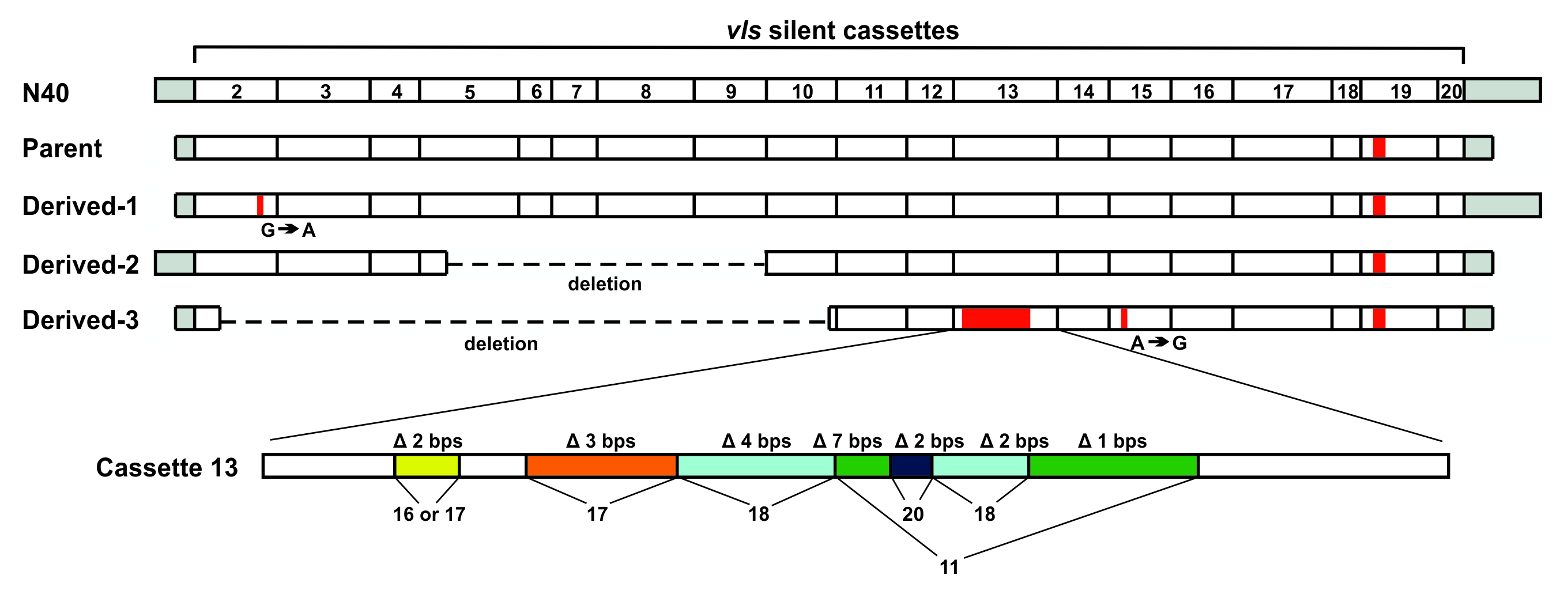

Supplement: Figure S4 — Experimental evolution of B. burgdorferi reveals mutations caused by gene conversions among unexpressed cassettes. Evolutionary changes observed in the cassette regions during experimental infections are characterized primarily by gene-conversions and large deletions. The parental N40 isolate differs from a previously sequenced N40 laboratory isolate in a 70 bp segment of cassette 19 which contains 12 substitutions and a 6 bp deletion. These sequence changes are identical to the homologous region of cassette 20 and are shared in all derived strains. Mutations that occurred during infection consisted of gene conversion events in derived isolates 1 and 3 and large deletions in derived isolates 2 and 3. The mutations in cassette 13 of derived isolate 3 are depicted with different colors to represent gene conversion from different donor cassettes. At least 6 overlapping gene conversion events from other cassettes are necessary to account for the mutations that occurred in this cassette. Of the two single-nucleotide substitutions in cassettes occurred during experimental evolution, the substitution that occurred in an antigenically important region (cassette 2 of derived isolate 1) was non-synonymous, whereas the substitution that occurred in a alpha helical region (cassette 15 of derived isolate 3) was synonymous. (TIF) [file ppat.1003766.s004.tif]

Derived-2

Derived-3

Derived-1

Parent

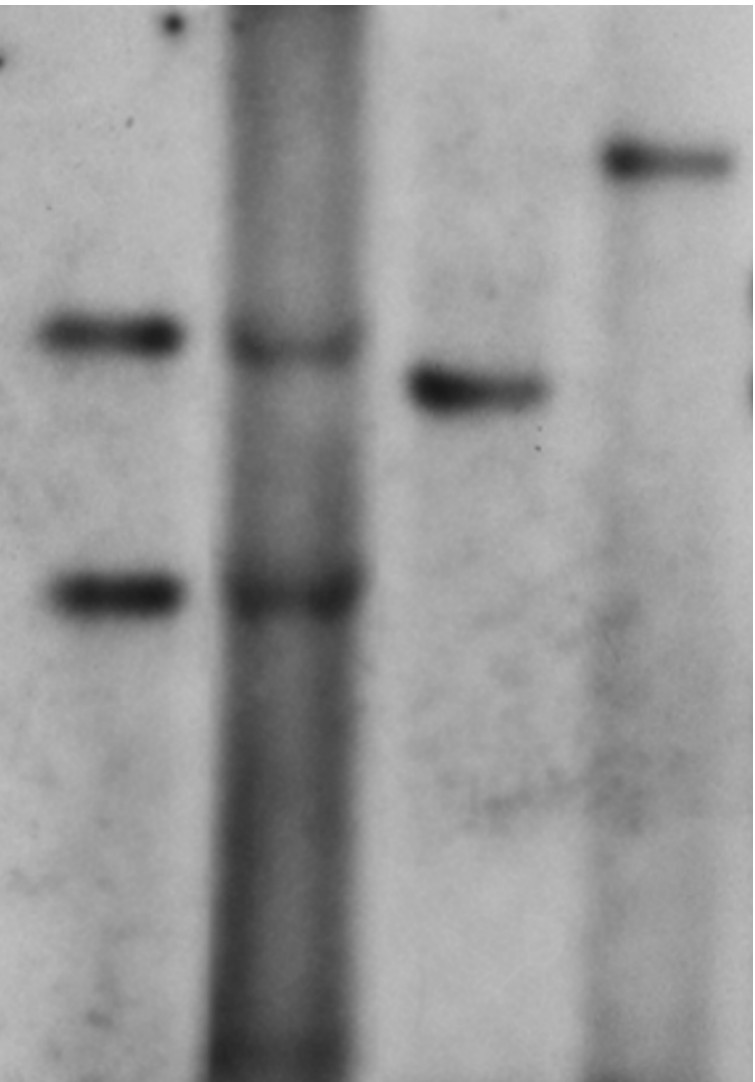

5.9kb →

4.5kb →

2.9kb →

Supplement: Figure S5 — Southern hybridization confirms large deletions in the vls cassette region. The large deletions observed in the unexpressed cassette region of derived strains 2 and 3 (Fig. S4) were confirmed by Southern blot of genomic DNA isolated from the B. burgdorferi strains. Genomic DNA was purified from the parental strain and each of the three derived strains as described in the text and digested with DraI endonuclease (NEB) which cuts at the recognition site, TTTAAA. Recognition sites for this enzyme occur throughout the AT rich genome of B. burgdorferi but are present at only one site within the vls unexpressed cassette region of each strain. The digested DNA was separated for four hours on a 0.4% agarose gel, transferred to a nylon membrane, and allowed to hybridize with two fluorescein labeled probes specific to the vls cassette sequences (5′ Fluor- TTC GGT TRG TGG KGA GCA GA 3′ and 5′ Fluor- ACA ATC CCC TTC ATC CCC TTA 3′). Southern blot analysis revealed two fragments (∼3 kb and ∼4.5 kb) in both the parental strain (cN40) and derived isolate 1 (36B) consistent with the single DraI site expected 2949 bp from the beginning of cassette 1 of a 7.467 kb fragment given the sequence data obtained in this study. The single fragment (∼4.3 kb) apparent in derived isolate 3 (39B) is consistent with the single DraI site expected 4204 bp from the beginning of cassette 1 of a 4.381 kb fragment. The 177 bp fragment was likely not detected because it ran off the gel. Similarly, derived isolate 2 (44B) revealed only a single fragment (∼5.9 kb) which is consistent with the single expected DraI site 5893 bp from the beginning of cassette 1 in a 6.073 kb fragment. (PDF) [file ppat.1003766.s005.pdf]
