# Supplementary material for: Natural Selection Promotes Antigenic Evolvability
Source: PLoS Pathog. 2013 Nov 14;9(11):e1003766. doi: 10.1371/journal.ppat.1003766 (PMC3828179; doi:10.1371/journal.ppat.1003766)
Supplement: Table S1 — Summaries of the silent cassette loci in B. burgdorferi strains analyzed in this study. (DOC) [file ppat.1003766.s006.doc]

**Table S1.** Summaries of the silent cassette loci in *B. burgdorferi* strains analyzed in this study.

| Genomic Isolate | Number of Cassettes | Genbank | Plasmid | Origin | AA Length (min,max,avg) | Frameshifts/ stop codons |
| --- | --- | --- | --- | --- | --- | --- |
| B31-5A3 | 15 | U76406 | lp28-1 | Tick, USA (NY) | 158 , 198, 183 | 2/1 |
| Bol26 | 14 | NC_012497 | lp28-3 | Human, Italy | 72 256 176 | 4/0 |
| JD1 | 14 | CP002306 | lp28-1 | Tick, USA (MA) | 103, 223, 190 | 0/0 |
| N40 | 19 | CP002230 | lp36 | Tick, USA (NY) | 56, 228, 131 | 0/1 |
| 72a | 3* | NC_011966 | lp32-3 | Human, USA (NY) | NA | 0/0 |
| 118a | 11* | CP001530 | lp32-3 | Human, USA (NY) | 79, 194, 138 | 1/0 |
| 297 | 11* | CP002265 | lp28-1 | Human, USA (CT) | 61, 236, 180 | 2/0 |
| 156a | 17* | CP001273 | lp28-1 | Human, USA (NY) | 80, 214, 178 | 0/0 |
| 64b | 22 | CP001423 | lp28-1 | Human, USA (NY) | 146, 195, 184 | 4/0 |
| WI91-23 | 16 | CP001456 | lp28-1 | Bird, USA (WI) | 27, 257, 185 | 2/0 |
| 94a | 6* | NZ ABGK02000011 | lp28-8 | Human, USA (NY) | 183, 207, 197 | 0/0 |
| ZS7 | 12* | NC_011780 | lp28-1 | Tick, Germany | 56, 199, 173 | 4/0 |
| 29805 | 18 | CP001557 | lp36 | Tick, USA (CT) | 88, 314, 180 | 0/0 |

* Incomplete sequence of cassette locus, number of identifiable cassettes in available sequence is provided

Summary of the *vls* silent sequences in the *B. burgdorferi* strains analyzed in our study. Strains vary in the number and length of unexpressed sequences as well as the linear plasmid on which the cassettes are contained. Strain 72a contains only two complete unexpressed cassettes in the reported sequence and was utilized only in the phylogenetic analysis.
