# Supplementary material for: Natural Selection Promotes Antigenic Evolvability
Source: PLoS Pathog. 2013 Nov 14;9(11):e1003766. doi: 10.1371/journal.ppat.1003766 (PMC3828179; doi:10.1371/journal.ppat.1003766)
Supplement: Table S2 — Statistical analysis of positive selection within silent cassettes of B. burgdorferi strains. (DOC) [file ppat.1003766.s007.doc]

**Table S2.** **Statistical analysis of positive selection within unexpressed cassettes of *B.* *burgdorferi* strains.**

|  | **α-Helical Regions** | | | **Antigenic Loop Regions** | | | **Z Test** | |
| --- | --- | --- | --- | --- | --- | --- | --- | --- |
| **Strain** | **dN** | **dS** | **dN/dS** | **dN** | **dS** | **dN/dS** | **Z Stat** | **p-value** |
| **B31** | 0.01 | 0.03 | 0.52 | 0.17 | 0.04 | 5.54 | 3.335 | < 0.001 |
| **N40** | 0.02 | 0.03 | 0.59 | 0.21 | 0.06 | 4.16 | 4.710 | < 0.001 |
| **JD1** | 0.05 | 0.05 | 1.11 | 0.22 | 0.12 | 2.08 | 1.811 | 0.036 |
| **Bol26** | 0.04 | 0.04 | 0.87 | 0.16 | 0.08 | 2.29 | 1.741 | 0.042 |
| **94a** | 0.03 | 0.04 | 1.04 | 0.26 | 0.09 | 3.07 | 3.048 | 0.001 |
| **118a** | 0.02 | 0.03 | 0.81 | 0.19 | 0.06 | 4.77 | 2.717 | 0.004 |
| **297** | 0.06 | 0.04 | 1.45 | 0.21 | 0.09 | 2.89 | 2.680 | 0.004 |
| **156a** | 0.05 | 0.06 | 1.10 | 0.21 | 0.12 | 2.17 | 1.636 | 0.052 |
| **29805** | 0.02 | 0.02 | 0.73 | 0.30 | 0.08 | 8.02 | 4.802 | < 0.001 |
| **64b** | 0.01 | 0.02 | 0.33 | 0.19 | 0.07 | 4.17 | 2.649 | 0.005 |
| **ZS7** | 0.01 | 0.03 | 0.54 | 0.20 | 0.04 | 5.63 | 2.917 | 0.002 |
| **WI91-23** | 0.01 | 0.03 | 0.40 | 0.18 | 0.09 | 2.47 | 0.500 | 0.309 |

The ratio of non-synonymous mutations per non-synonymous site (dN) and the ratio of synonymous differences per synonymous site (dS) reveals a substantial excess of non-synonymous polymorphisms in antigenically important loop regions of the silent sequence repertoires. The Z-test of positive selection over entire codon aligned cassette sequences was statistically significant in 10 of 12 of the strains. This is driven by a dramatic increase in dN (compared to dS) in the antigenic loop regions of the unexpressed cassette sequences, which is further supported by our codon-by-codon analyses (Fig. 3). Antigenic loop regions also have an increased proportion of synonymous differences in all strains, consistent with the observation of high frequencies of template-independent sequence changes to these regions of *vlsE* during infection (Coutte et al. 2009).
