# Supplementary material for: Natural Selection Promotes Antigenic Evolvability
Source: PLoS Pathog. 2013 Nov 14;9(11):e1003766. doi: 10.1371/journal.ppat.1003766 (PMC3828179; doi:10.1371/journal.ppat.1003766)
Supplement: Table S3 — Genbank accession numbers of the vls cassette region, ospA and IGS loci sequenced in isolates after one year of experimental infection. (DOC) [file ppat.1003766.s008.doc]

**Table S3.** Genbank accession numbers of the *vls* cassette region, *ospA* and *IGS* loci sequenced in isolates after one year of experimental infection.

| **Sample** | **Isolate*** | **Locus** | **Genbank** |
| --- | --- | --- | --- |
|  |  | *vls* cassettes | JN413100 |
| **Parent** | cN40 | *ospA* | JN413096 |
|  |  | *IGS* | JN413092 |
|  |  | *vls* cassettes | JN413101 |
| **Derived 1** | 36B | ospA | JN413097 |
|  |  | IGS | JN413093 |
|  |  | *vls* cassettes | JN413102 |
| **Derived 2** | 44B | *ospA* | JN413098 |
|  |  | *IGS* | JN413094 |
|  |  | *vls* cassettes | JN413103 |
| **Derived 3** | 39B | *ospA* | JN413099 |
|  |  | *IGS* | JN413095 |

*Name of re-isolate used in Stevenson et al. 1994
